# Oxidative Stress and Gene Expression Modifications Mediated by Extracellular Vesicles: An In Vivo Study of the Radiation-Induced Bystander Effect

**DOI:** 10.3390/antiox10020156

**Published:** 2021-01-21

**Authors:** Rita Hargitai, Dávid Kis, Eszter Persa, Tünde Szatmári, Géza Sáfrány, Katalin Lumniczky

**Affiliations:** Radiation Medicine Unit, Department of Radiobiology and Radiohygiene, National Public Health Centre, 1221 Budapest, Hungary; hargitai.rita@osski.hu (R.H.); kis.david@osski.hu (D.K.); persa.eszter@osski.hu (E.P.); szatmari.tunde@osski.hu (T.S.); safrany.geza@osski.hu (G.S.)

**Keywords:** antioxidant enzymes, apoptosis, DNA damage repair, extracellular vesicles, gene expression, ionising radiation, lipid peroxidation, oxidative damage

## Abstract

Radiation-induced bystander effect is a biological response in nonirradiated cells receiving signals from cells exposed to ionising radiation. The aim of this in vivo study was to analyse whether extracellular vesicles (EVs) originating from irradiated mice could induce modifications in the redox status and expression of radiation-response genes in bystander mice. C57BL/6 mice were whole-body irradiated with 0.1-Gy and 2-Gy X-rays, and EVs originating from mice irradiated with the same doses were injected into naïve, bystander mice. Lipid peroxidation in the spleen and plasma reactive oxygen metabolite (ROM) levels increased 24 h after irradiation with 2 Gy. The expression of antioxidant enzyme genes and inducible nitric oxide synthase 2 (iNOS2) decreased, while cell cycle arrest-, senescence- and apoptosis-related genes were upregulated after irradiation with 2 Gy. In bystander mice, no significant alterations were observed in lipid peroxidation or in the expression of genes connected to cell cycle arrest, senescence and apoptosis. However, there was a systemic increase in the circulating ROM level after an intravenous EV injection, and EVs originating from 2-Gy-irradiated mice caused a reduced expression of antioxidant enzyme genes and iNOS2 in bystander mice. In conclusion, we showed that ionising radiation-induced alterations in the cellular antioxidant system can be transmitted in vivo in a bystander manner through EVs originating from directly irradiated animals.

## 1. Introduction

Exposure to ionising radiation (IR) can directly induce biochemical changes in living cells by damaging macromolecules (nucleic acids, proteins and lipids). In addition, it can cause indirect harm through the radiolysis of water and, thus, the generation of reactive oxygen and nitrogen species (ROS and RNS) [1,2,3]. Oxidative damage, including lipid peroxidation, protein carbonylation and oxidative DNA lesions, may continue to occur for days or months after the direct exposure to IR [2,4,5]. A change in the redox (antioxidant/pro-oxidant) balance towards free radical generation could lead to oxidative stress and diseases like cancer, degenerative diseases and premature aging [6,7,8,9].

It has been noted that not only cells directly hit by a radiation beam can be damaged after exposure to IR, but there are similar radiation-related changes in nonirradiated neighbouring or distant cells (“bystander effects”) or in the progeny of irradiated cells occurring generations after direct irradiation (“genomic instability”) [1,10,11,12]. The radiation-induced bystander effect (RIBE) is defined as a biological response developing in nonirradiated cells receiving signals from other cells directly exposed to IR [13]. The manifestations of RIBE are very diverse, consisting of an increased frequency of DNA damage (e.g., mutations, DNA double-strand breaks (DSBs) and increased micronuclei formation); chromosomal aberrations; modified gene and protein expressions; epigenetic alterations; oncogenic transformation; increased cell death and oxidative stress [14,15,16,17,18,19,20].

RIBE has been extensively studied in the past decade, and great efforts have been made to understand the mechanisms regulating it [12,21,22,23,24]. Nonetheless, the mechanisms of RIBE are still unclear, and they are likely to be complex, involving multiple pathways [25]. Intercellular communication between directly hit and bystander cells could be mediated by several pathways, including cell–cell contact (gap junctions); the transfer of secreted soluble factors (cytokines, nitric oxide and ROS) and signal molecules carried by extracellular vesicles (EVs) [15,19,26,27,28,29]. EVs are nanovesicles actively released by most cell types into body fluids and taken up by target cells [30]. EVs transfer various nucleic acids (DNA fragments, mitochondrial DNA, mRNA and microRNA); proteins and lipids originating from the donor cells to specific target cells to regulate downstream signalling pathways [31,32], and the content of EVs of irradiated cells is different compared to EVs of nonirradiated cells [28,33,34,35,36]. EVs can modulate the gene expression through the delivered mRNA or via post-transcriptional regulation by microRNAs (miRNAs) [28,31,36].

The antioxidant defence system acts through several pathways against the produced ROS, including endogenous, synthesised antioxidant enzymes and nonenzymatic molecules, as well as exogenous, ingested antioxidants [2,3]. If this defence network works insufficiently, then oxidative stress appears. Superoxide dismutase (SOD) is a metal-containing (Cu/Zn or Mn) protein that catalyses the removal of superoxides, producing hydrogen peroxides [37,38]. It was shown that irradiation resulted in a dose-dependent decline in SOD activity, which led to increased lipid peroxidation [39]. Catalase (CAT) is a heme-containing enzyme, which catalyses the conversion of hydrogen peroxide to oxygen and water [38]. CAT was shown to abrogate RIBE-induced DNA damage response in human fibroblast cells [40]. The function of glutathione-s-transferase (GST) is the detoxification of endogenous and exogenous electrophilic compounds (e.g., aldehydes and ketones) by catalysing the conjugation of the reduced form of glutathione to them [41].

To further cope with the damaging effects of ROS and RNS, the cell cycle is arrested, and DNA repair mechanisms are employed, or, in the case of severe damage, cells are removed by apoptosis. In several studies, BCL2-binding component 3 (BBC3), growth arrest and DNA-damage-inducible 45 alpha (GADD45a) and cyclin-dependent kinase inhibitor 1A (CDKN1A or p21) genes were identified as radiation response genes in studies of human and murine peripheral blood lymphocytes [42,43,44]. BBC3 is upregulated due to diverse stress stimuli; it is a proapoptotic protein that can initiate the onset of apoptosis [45]. CDKN1A plays a regulatory role in DNA replication and DNA damage repair [46]. When cell cycle arrest is needed to allow for DNA damage repair, p53 induces CDKN1A to inhibit the progression of the cell cycle [47]. GADD45a responds to environmental stresses by mediating the activation of the P38/JNK pathway, and it is a cell cycle regulator [48]. The ataxia telangiectasia mutated (ATM) gene is activated by DNA DSBs, and it plays an important role in initiating the DNA damage repair signalling pathway [49,50]. Numerous stressful stimuli, including oxidative stress and DNA damage, lead to the onset of senescence, which is a state of permanent cell cycle arrest [51]. Cyclin-dependent kinase inhibitor 2A (CDKN2A or p16) is a cell cycle inhibitor [52,53]. Its expression is upregulated in senescing cultured cells, and it is considered to have important roles in establishing the senescent state [52,54].

Cyclooxigenase-2 (COX-2) and inducible nitric oxide synthase 2 (iNOS2) may play important roles in RIBE [17,55]. COX-2 can be induced by various growth factors, cytokines, inflammatory signals and several stress factors [56]. It was found that bystander cells showed an overexpression of the COX-2 gene, and the suppression of COX-2 activity in bystander cells resulted in a significantly reduced bystander effect [17,55,57]. It was reported that tissues exposed to IR or inflammatory insults (cytokines and oxidative xenobiotics) showed increased iNOS2 activity [56,58]. iNOS2 produces large quantities of nitric oxide (NO) [59], which could be responsible for bystander effects as well, as it can damage DNA and DNA repair proteins, and it can diffuse freely into cells [26,60,61].

Earlier studies evaluated the possible role of oxidative stress in the induction of RIBE. ROS appeared to be major contributors to the activation of several stress-inducible signalling pathways, as well as micronucleus formation in bystander cells, and it was found that antioxidant enzymes inhibited the induction of RIBE in bystander cells [2,40,62,63]. Although RIBE has been extensively studied, still few studies have investigated the role of EVs in mediating oxidative stress in bystander cells. In vitro, it was found that EVs isolated from cancer cell cultures were able to activate proteins involved in the DNA damage response (ATM, H2AX and p53) by elevating ROS production in EV-recipient cells [64]. A recent review showed indications for the role of transmitting signals carried by EVs in cellular redox biology [65]. EVs were shown to contain various proteins important for redox processes (e.g., GST, SOD, NOS3 and NADPH oxidase) [66,67,68,69,70] and oxidised DNA fragments [71,72], and EVs may exhibit intrinsic ROS production [68,73]. An ex vivo study showed that EVs from preeclamptic women induced the upregulation of iNOS2 and COX-2 expression and activation of the transcription factor nuclear factor kappa B (NF-κB) in endothelial cells, enhancing oxidative stress [57]. However, to our knowledge, no in vivo study has investigated so far whether EVs isolated from irradiated tissues could induce oxidative stress and modify the related gene expression in bystander tissues.

In our previous study, we found that bone marrow-derived EVs of irradiated mice induced bystander effects in the bone marrow and spleen of nonirradiated recipient mice [28]. Eight miRNAs were identified that were differentially expressed in the EVs of both 0.1-Gy- and 2-Gy-irradiated mice as compared to EVs of nonirradiated mice [28]. The most enriched pathways of this set of miRNAs involved pathways related to hematopoietic and immune system regulation; oxidative stress resistance; cell cycle regulation and apoptosis (e.g., the FoxO signalling pathway, Hippo signalling pathway, TGF-beta signalling pathway and Wnt signalling pathway) [28]. EVs induced the activation of the DNA damage response, detected by γ-H2AX foci formation in spleen cells [28], one of the earliest cellular responses to DNA DSBs [74,75]. Increased γ-H2AX foci formations in bystander cells were observed in several other in vitro and in vivo models [18,28,29,76], and it was suggested that imbalanced redox homeostasis could be the underlying cause of it [28,76]. The aim of our present study was to analyse whether bystander responses initiated by the transmission of EVs originating from mice irradiated with low- and high-dose radiation could induce oxidative damage and modifications in the expression of genes involved in oxidative stress regulation and other pathways specifically altered by ionising radiation.

## 2. Materials and Methods

### 2.1. Animals

Ten–thirteen-week-old C57BL/6 male mice were used for the experiments. Mice were kept under standard nursery conditions. Animal studies were approved by the National Scientific Ethical Committee on Animal Experimentation (identification number: KA 2113), and permission was issued by the Food Chain Safety and Animal Health Directorate of the Government Office of Pest County (permit number: PE/EA/392-7/2017 and PEI/001/1734-4/2015).

### 2.2. Irradiation and Sample Collection

Mice were whole-body irradiated with the following doses: 0 Gy (control, sham irradiated), 0.1 Gy (7.59 cGy/min) and 2 Gy (80 cGy/min) using a X-RAD 225XL (Precision X-ray, Inc. North Branford, CT, USA) X-ray source. For each dose, 6 or 7 mice were used. Twenty-four hours after irradiation, mice were euthanised, and tissue samples (blood, spleen and bone marrow) were collected. Blood (ca. 400–600 μL) was collected from the hepatic portal vein into a tube containing 50 μL of sodium heparin (Richter-Gedeon Rt., Budapest, Hungary) as an anticoagulant. Plasma was separated by centrifugation at 2000× *g* at 4 °C for 10 min and stored at −75 °C until later analyses. Spleens were snap-frozen in liquid nitrogen, and the frozen spleens were cut into pieces and stored at −75 °C for later analyses.

### 2.3. EV Isolation, Characterisation and Bystander Experiment

Bone marrows of directly irradiated mice were isolated from the femur and tibia by flushing out the tissue to 0.5 mL of phosphate-buffered saline (PBS) 24 h following irradiation. Bone marrow supernatants of mice irradiated with the same dose were pooled. Cells were pelleted by centrifugation at 400× *g* for 10 min at 4 °C. EVs were isolated from bone marrow supernatant with the ExoQuick-TC kit (System Biosciences, Palo Alto, CA, USA), following the manufacturer’s instructions (see, for details, [28]). The quality of EVs isolated with the applied methods was assessed by several techniques, as previously presented in our works [28,77]. The presence of EV-related molecules (inner protein TSG101 and surface protein CD9) was evaluated with Western blot analysis [28]. EV size and morphology was assessed by the dynamic light scattering (DLS) method and transmission electron microscopy [28,77]. EV protein concentration was determined by the Bradford protein assay kit (Thermo Scientific, Waltham, MA, USA) using a microplate reader (Synergy HT, BioTek, Winooski, VT, USA) [28,33,77].

Ten micrograms of EVs (based on the EV protein concentration) isolated from 0-Gy-, 0.1-Gy- or 2-Gy-irradiated mice were suspended in 140 μL of 100-nm-filtered PBS and injected into the tail veins of nonirradiated naïve (“bystander”) mice of the same age. Blood and spleen from the bystander mice were isolated 24 h after EV injection, similar to directly irradiated mice, as described above.

### 2.4. Lipid Peroxidation

Proteins were extracted from 30–40 mg frozen spleen samples applying a Dounce tissue homogeniser with 200 μL of PBS and 2 μL of a Halt Protease inhibitor cocktail (Thermo Scientific, Waltham, MA, USA). Protein concentration was measured with the Bradford protein assay kit (Thermo Scientific, Waltham, MA, USA). Lipid peroxidation in the protein extract was analysed with the Oxiselect HNE-Adduct competitive ELISA assay kit (Cell BioLabs, Inc., San Diego, CA, USA), following the manufacturer’s instructions. 4-hydroxy-trans-2-nonenal (4-HNE) is one of the most abundant and relatively stable products of lipid peroxidation, and it has been used as a biomarker for the identification of acute and chronic oxidative stress following irradiation [4,5]. Standards and samples were measured in duplicates. Absorbance of each well was measured with a microplate reader (Synergy HT, BioTek, Winooski, VT, USA) at 450 nm. Lipid peroxidation (HNE-adduct concentration) was calculated using a calibration curve of HNE-BSA standards (supplied by the manufacturer), and it was expressed as ng/μg protein.

### 2.5. Plasma Oxidative Damage

Plasma levels of reactive oxygen metabolites (ROMs, primarily hydroperoxides) were measured with the d-ROM assay (Diacron International, Grosseto, Italy), following the manufacturer’s instructions. ROMs are intermediate oxidative damage compounds, which are generated by the peroxidation of macromolecules (nucleic acids, lipids and proteins) by ROS. ROMs are more stable than ROS, and therefore, they can be detected and quantified [78,79,80]. Five microlitres of plasma were added to 195 μL of a solution containing a chromogen (*N*,*N*-diethyl-p-phenylenediamine) (R1 reagent) and acetate buffer (R2 reagent) in a 1:100 ratio and incubated at 37 °C for 85 min with continuous shaking. Iron and copper ions were released from plasma proteins in the presence of the acidic R2 reagent (an acetate buffer), and alkoxyl and peroxyl radicals were generated from hydroperoxides. These highly reactive compounds oxidised the chromogen and transformed it into a pink derivative. The intensity of the pink colour was proportional to the concentration of ROMs in the sample. Absorbance of each well was read with a microplate reader (Synergy HT, BioTek, Winooski, VT, USA) at 505 nm. ROM concentration was calculated using a calibration curve of lyophilised serum standard supplied by the manufacturer. Standards and samples were measured in duplicates. Plasma ROM concentration was expressed as mg of hydrogen peroxide equivalents/dL, where 1 arbitrary unit called “Carratelli units” corresponded to 0.08 mg of hydrogen peroxide equivalents/dL.

### 2.6. Gene Expression

Total RNA was extracted with the Qiagen RNeasy Mini Kit (Qiagen, Hilden, Germany), following the manufacturer’s instructions. Frozen spleen samples (10–20 mg) were added to 200 μL of ice cold RLT buffer (a lysis buffer of the RNeasy Mini Kit) with 1% β-mercaptoethanol (BME) and disrupted in a Dounce tissue homogeniser. An additional 550 μL of RLT buffer (with 1% BME) was added to the lysate; then, it was further homogenised by using a 20-G needle. DNA was eliminated using the RNase-free DNase Set (Qiagen, Hilden, Germany). Total RNA was eluted with 30 μL of RNase-free water and stored at −20 °C until later analysis. RNA quality was assessed by agarose gel electrophoresis.

One microgram of total RNA was used for reverse transcription to produce cDNA using the RevertAid RT Reverse Transcription Kit (Thermo Scientific, Waltham, MA, USA), according to the manufacturer’s instructions. For the gene expression evaluation, real-time PCR analyses were performed with the Maxima SYBR Green qPCR master mix (Thermo Scientific, Waltham, MA, USA). The polymerase (RNA) II (DNA-directed) polypeptide G (POLR2G) gene was applied as a housekeeping gene. The sequences of the oligonucleotide primers (Integrated DNA Technologies, Leuven, Belgium) are shown in the Supplementary Materials (Table S1); the final concentration of the primers was 0.6 μM. The cycling conditions were as follows: hot start at 95 °C for 10 min, then 40 cycles at 95 °C for 15 s, 60 °C for 30 s and elongation at 72 °C for 30 s. Amplification procedures were carried out in a Rotor-Gene Q (Qiagen, Hilden, Germany) real-time PCR thermocycler; all samples were run in triplicates. The specificity of the PCR amplicons was assessed by the melting curve inspection and agarose gel electrophoresis (Supplementary Materials, Figure S1). Delta-delta cycle threshold (Ct) values of directly irradiated mice were calculated for each gene using the average delta Ct values of 0-Gy mice (*n* = 6) for the given gene, while those of bystander mice were calculated using the average delta Ct values of 0-Gy bystander mice (*n* = 3 or 6) for the given gene.

### 2.7. Statistical Analyses

Data were analysed with two-tailed, unpaired Student’s *t*-tests to compare the values of 0.1-Gy and 2-Gy vs. 0-Gy and the values of 0.1-Gy bystander and 2-Gy bystander vs. 0-Gy bystander. We used F-tests to compare the variances between the groups analysed with *t*-tests, and when they were significant (*p* < 0.05), unpaired *t*-tests with Welch’s correction were performed. Statistical results with *p*-values lower than 0.05 were considered significant. Data are presented as mean ± standard deviation (SD). Statistical analyses were performed by GraphPad Prism, version 6.04 (GraphPad Software, La Jolla, CA, USA).

## 3. Results

### 3.1. Effects of Direct Irradiation and EV-Transfer from Irradiated Mice on Lipid Peroxidation in the Spleen

**Figure 1:**
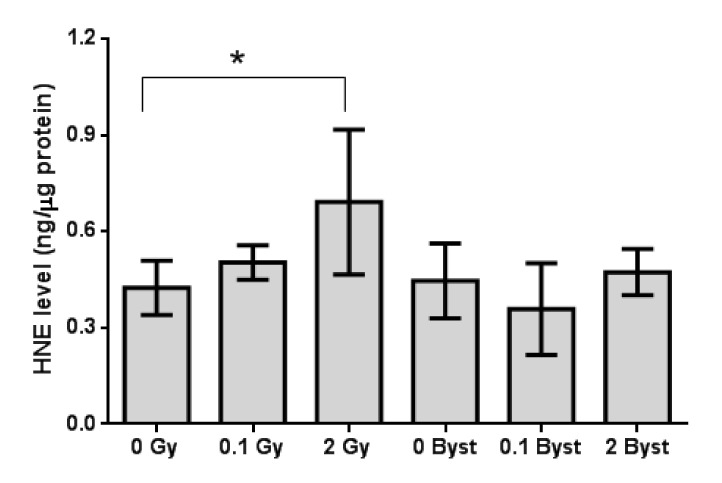
Lipid peroxidation based on the hydroxy-trans-2-nonenal (HNE)-adduct concentration (ng/μg protein) of the spleen tissues of mice 24 h after direct irradiation (0 Gy, 0.1 Gy and 2 Gy) or extracellular vesicle (EV) transfer from irradiated mice (0-Gy bystander, 0.1-Gy bystander and 2-Gy bystander). The HNE-adduct concentration was determined using the Oxiselect HNE-Adduct competitive ELISA assay kit (see Materials and Methods for details). Data were analysed with unpaired Student’s *t*-tests; * *p* < 0.05. Bars and whiskers represent mean ± SD (*n* = 6).

Lipid peroxidation in the spleen of mice irradiated with 2 Gy was moderately (1.6-fold) increased as compared to the nonirradiated mice (*p* = 0.033), and there was also a small, marginally significant (1.2-fold) increase (*p* = 0.085) in lipid peroxidation in the spleen of mice irradiated with 0.1 Gy (Figure 1). In bystander mice, no significant difference was observed (all *p* > 0.27) between mice injected with EVs from nonirradiated mice and from 0.1-Gy- or 2-Gy-irradiated mice (Figure 1). Lipid peroxidation in the spleen showed no significant difference between 0 Gy and 0-Gy bystander mice (*p* = 0.72).

### 3.2. Effects of Direct Irradiation and EV-Transfer from Irradiated Mice on the Plasma Level of Oxidative Damage

**Figure 2:**
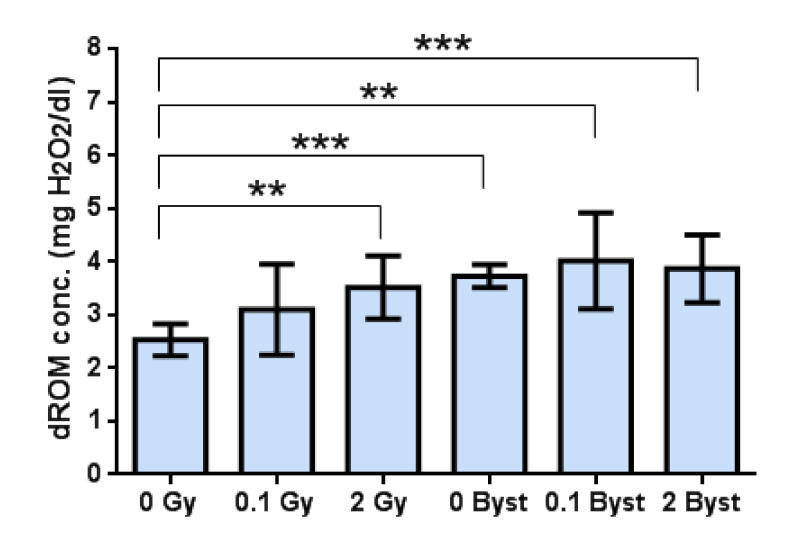
Plasma level of derivates of reactive oxygen metabolites (dROM) (mg H_2_O_2_/dL) of mice 24 h after direct irradiation (0 Gy, 0.1 Gy and 2 Gy) or EV transfer from irradiated mice (0-Gy bystander, 0.1-Gy bystander and 2-Gy bystander). The plasma dROM concentration was measured with the dROM assay (see Materials and Methods for details). Data were analysed with unpaired Student’s *t*-tests; ** *p* < 0.01 and *** *p* < 0.001. Bars and whiskers represent mean ± SD (*n* = 6).

Systemic oxidative damage (evaluated by the level of ROMs in the plasma) changed very similarly to lipid peroxidation in the spleen. Namely, low-dose direct irradiation with 0.1 Gy induced no significant changes (*p* = 0.17), while irradiation with 2 Gy induced a moderate (1.4-fold) increase (*p* = 0.004) in the level of plasma ROMs (Figure 2). In bystander mice, no significant difference (all *p* > 0.48) was observed between mice injected with EVs from nonirradiated mice and from 0.1-Gy- or 2-Gy-irradiated mice (Figure 2). However, we found that the plasma level of oxidative damage in all bystander mice was significantly higher than in 0-Gy (control) mice (all *p* < 0.01) (Figure 2).

### 3.3. Effects of Direct Irradiation and EV Transfer from Irradiated Mice on the Antioxidant and Pro-Oxidant Gene Expressions

**Figure 3:**
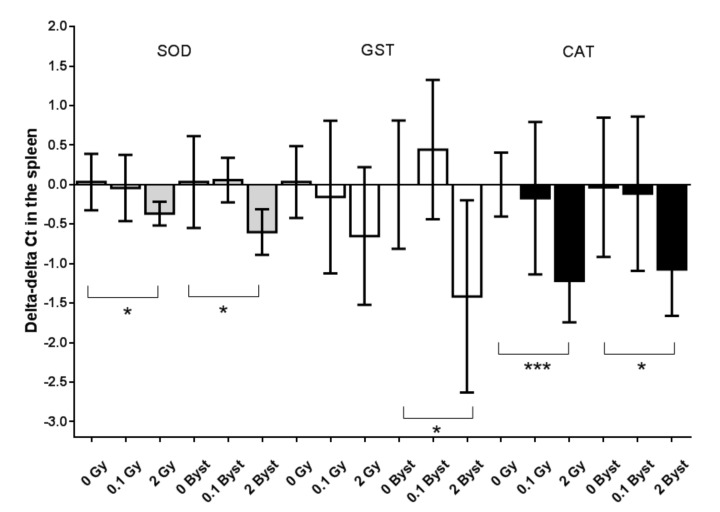
Relative changes in the gene expressions (indicated as delta-delta cycle threshold (Ct) values) of superoxide dismutase (SOD, grey), glutathione-s-transferase (GST, white) and catalase (CAT, black) of the spleen tissues of mice 24 h after direct irradiation (0 Gy, 0.1 Gy and 2 Gy) or EV transfer from irradiated mice (0-Gy bystander, 0.1-Gy bystander and 2 Gy bystander) in relation to the average delta Ct value of the 0 Gy (*n* = 6) or 0-Gy bystander groups (*n* = 6), respectively. The gene expressions were evaluated with real-time PCR by applying polymerase (RNA) II (DNA-directed) polypeptide (POLR2G) as a housekeeping gene. +1 delta–delta Ct values indicate a 2-fold relative gene expression, while −1 delta-delta Ct values indicate a 0.5-fold relative gene expression compared to nonirradiated mice (see Materials and Methods for details). Data were analysed with unpaired Student’s *t*-tests; * *p* < 0.05 and *** *p* < 0.001. Bars and whiskers represent mean ± SD (*n* = 6 or 7).

The expressions of SOD2 and CAT in the spleen were significantly lower 24 h after irradiation with 2 Gy than those of the nonirradiated mice (SOD2: 0.8-fold change; *p* = 0.030 and CAT: 0.4-fold change; *p* = 0.001) (Figure 3). The expression of GST did not change significantly after 2-Gy irradiation (*p* = 0.12) (Figure 3). Low-dose (0.1 Gy) irradiation caused no modification in the expression of any of the antioxidant enzymes in the spleen (all *p* > 0.64) (Figure 3). The expressions of all three antioxidant enzymes were significantly lower in bystander mice injected with EVs from 2-Gy-irradiated mice than in those injected with EVs from nonirradiated mice (SOD2: 0.6-fold change; *p* = 0.027, CAT: 0.5-fold change; *p* = 0.028 and GST: 0.4-fold change; *p* = 0.034). Similar to direct irradiation, injection with EVs from 0.1-Gy-irradiated mice elicited no significant modifications in the expressions of the studied antioxidant enzymes (all *p* > 0.37) (Figure 3).

**Figure 4:**
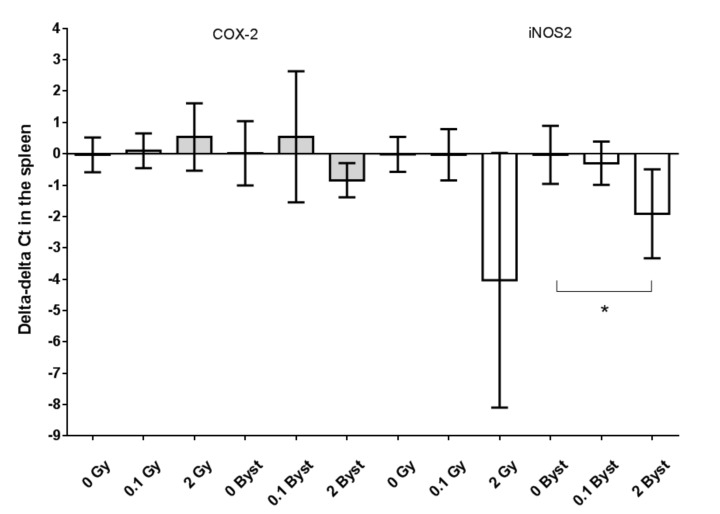
Relative changes in the gene expressions (indicated as delta-delta cycle threshold (Ct) values) of cyclooxigenase-2 (COX-2, grey) and inducible nitric oxide synthase 2 (iNOS2, white) of the spleen tissues of mice 24 h after direct irradiation (0 Gy, 0.1 Gy and 2 Gy) or EV transfer from irradiated mice (0-Gy bystander, 0.1-Gy bystander and 2-Gy bystander) in relation to the average delta Ct values of the 0 Gy (*n* = 6) or 0-Gy bystander groups (*n* = 6), respectively. The gene expressions were evaluated with real-time PCR by applying polymerase (RNA) II (DNA-directed) polypeptide (POLR2G) as a housekeeping gene. +1 delta–delta Ct values indicate a 2-fold relative expression, while −1 delta-delta Ct values indicate a 0.5-fold relative expression compared to nonirradiated mice (see Materials and Methods for details). Data were analysed with unpaired Student’s *t*-tests; * *p* < 0.05. Bars and whiskers represent mean ± SD (*n* = 6 or 7).

We found that the expression of COX-2 in the spleen did not change significantly either in directly irradiated (all *p* > 0.28) or in bystander mice (all *p* > 0.11) (Figure 4). The expression of iNOS2 showed a marginally significant decline 24 h after 2-Gy irradiation (0.1-fold change; *p* = 0.060; Figure 4) but not after 0.1-Gy irradiation (*p* = 0.98). The expression of iNOS2 was significantly lower in mice injected with EVs from 2-Gy-irradiated mice than in mice injected with EVs from nonirradiated mice (0.3-fold change; *p* = 0.018) but not in 0.1-Gy bystander mice (*p* = 0.56) (Figure 4).

The expression of the studied antioxidant and pro-oxidant genes relative to the expression of the housekeeping gene (expressed as delta Ct values) showed no significant differences between 0 Gy and 0-Gy bystander mice spleen tissues (all *p* > 0.15).

### 3.4. Effects of Direct Irradiation and EV Transfer from Irradiated Mice on the Cell Cycle, Senescence and Apoptosis Regulator Gene Expression

**Figure 5:**
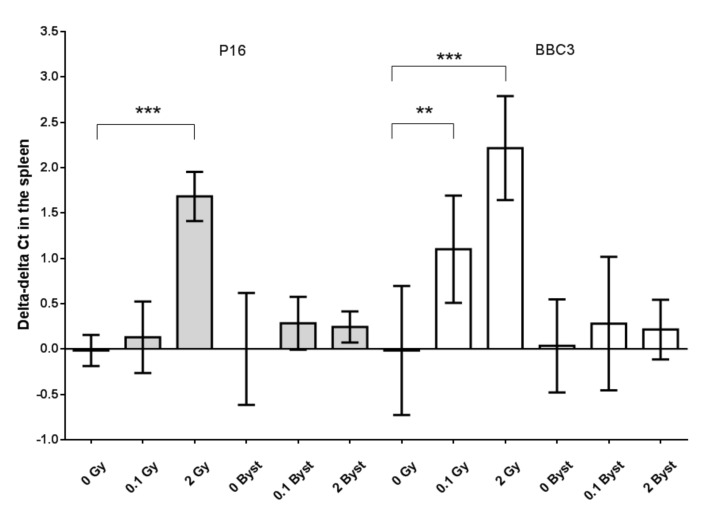
Relative changes in the gene expression (indicated as delta-delta cycle threshold (Ct) values) of cyclin-dependent kinase inhibitor 2A (P16, grey) and BCL2-binding component 3 (BBC3, white) of the spleen tissues of mice 24 h after direct irradiation (0 Gy, 0.1 Gy and 2 Gy) or EV transfer from irradiated mice (0-Gy bystander, 0.1-Gy bystander and 2-Gy bystander) in relation to the average delta Ct values of the 0 Gy (*n* = 6) or 0-Gy bystander groups (*n* = 6 or 3), respectively. The gene expressions were evaluated with real-time PCR by applying polymerase (RNA) II (DNA-directed) polypeptide (POLR2G) as a housekeeping gene. +1 delta–delta Ct values indicate a 2-fold relative expression, while −1 delta-delta Ct values indicate a 0.5-fold relative expression compared to nonirradiated mice (see Materials and Methods for details). Data were analysed with unpaired Student’s *t*-tests; ** *p* < 0.01 and *** *p* < 0.001. Bars and whiskers represent mean ± SD (P16: *n* = 6 or 7 and BBC3: *n* = 3–7).

**Figure 6:**
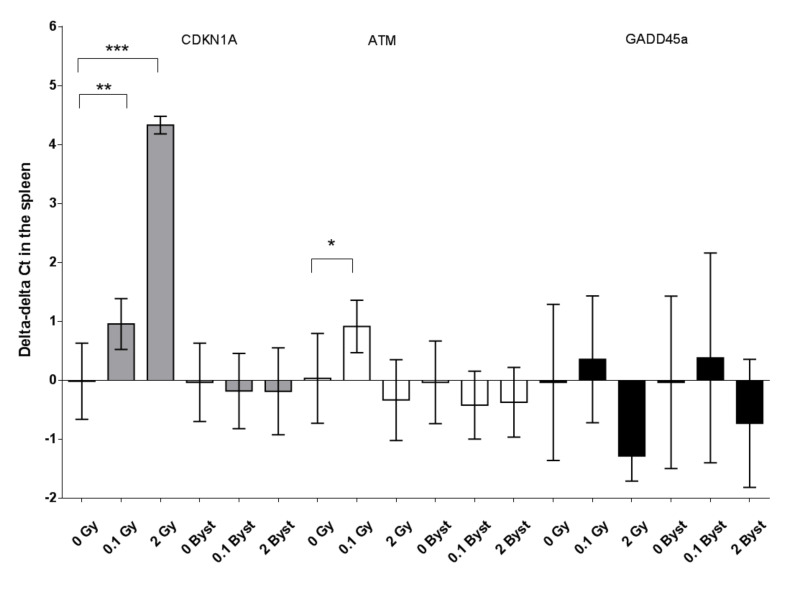
Relative changes in the gene expression (indicated as delta-delta cycle threshold (Ct) values) of cyclin-dependent kinase inhibitor 1A (CDKN1A, grey), ataxia telangiectasia mutated (ATM, white) and growth arrest and DNA-damage-inducible 45 alpha (GADD45a, black) of the spleen tissues of mice 24 h after direct irradiation (0 Gy, 0.1 Gy and 2 Gy) or EV transfer from irradiated mice (0-Gy bystander, 0.1-Gy bystander and 2-Gy bystander) in relation to the average delta Ct values of the 0 Gy (*n* = 6) or 0-Gy bystander groups (*n* = 3), respectively. The gene expressions were evaluated with real-time PCR applying polymerase (RNA) II (DNA-directed) polypeptide (POLR2G) as a housekeeping gene. +1 delta-delta Ct values indicate a 2-fold relative expression, while −1 delta-delta Ct values indicate a 0.5-fold relative expression compared to nonirradiated mice (see Materials and Methods for details). Data were analysed with unpaired Student’s *t*-tests; * *p* < 0.05, ** *p* < 0.01 and *** *p* < 0.001. Bars and whiskers represent mean ± SD (*n* = 3–7).

The expression of p16 in the spleen was significantly higher in mice directly irradiated with 2 Gy than in nonirradiated mice (3.2-fold change; *p* < 0.001) (Figure 5). The expression of BBC3 and CDKN1A showed dose-dependent increases in directly irradiated mice. BBC3 showed a 2.2-fold change (*p* = 0.010) after 0.1-Gy and a 4.7-fold change (*p* < 0.001) after 2-Gy irradiation (Figure 5). CDKN1A showed a 1.9-fold change (*p* = 0.008) after 0.1-Gy and a 19.5-fold change (*p* < 0.001) after 2-Gy irradiation (Figure 6). In the case of ATM, the expression increased only after low-dose irradiation (1.8-fold change; *p* = 0.025), while the GADD45A expression showed an almost significant decrease 24 h after 2-Gy irradiation (0.4-fold change; *p* = 0.070) (Figure 6). In bystander animals, none of these genes (p16, BBC3, CDKN1A, ATM and GADD45a) showed an altered expression 24 h after EV injection from 0.1-Gy- or 2-Gy-irradiated mice as compared to mice injected with EVs from nonirradiated mice (all *p* > 0.19) (Figure 5 and Figure 6). The expression of the studied genes relative to the expression of the housekeeping gene (expressed as delta Ct values) showed no significant difference between 0 Gy and 0-Gy bystander mice spleen tissues (all *p* > 0.27).

## 4. Discussion

It has been described in several in vivo studies that exposure to IR can cause oxidative damage to DNA, lipids and proteins in various tissues, indicated by the elevated formation of lipid peroxidation products, oxidative DNA products (8-OHdG) and protein carbonyls [3,81,82,83]. For example, increases in the 8-OHdG and 4-HNE levels were measured in the bone marrow and liver of rats after 3-Gy X-ray irradiation [84]. In vivo exposure to 10–20 Gy of fractioned γ-rays (2 Gy/day) resulted in increased lipid peroxidation, while SOD and glutathione peroxidase (GPx) activities showed a dose-dependent decline in the skin of mice [39]. Similar to our results, mice irradiated with high doses (7.5 and 14 Gy, respectively) showed elevated levels of ROMs in the plasma, which were reduced by pretreatment with antioxidants [79,85]. Radiation-treated oncological patients showed a marked increase in plasma levels of ROMs at the end of radiotherapy receiving 2-Gy fractions/day for five–seven weeks [86,87]. Moreover, it was reported that plasma levels of ROMs were higher in a songbird species breeding in sites with higher radiation levels in the Chernobyl region [78]. Although we used lower doses than other studies, our results also support these findings, as lipid peroxidation in the spleen and circulatory levels of ROMs were higher in mice irradiated with 2 Gy than in nonirradiated mice. It is also well-documented that high-dose irradiation inhibits multiple components of the cellular antioxidant system. For example, decreased SOD2 and CAT expression in splenocytes or other cell types 24 h after exposure to very high doses (6 Gy and 20 Gy, respectively) was reported by previous studies [88,89], and our present study also supports these findings when applying a much lower irradiation dose (2 Gy).

While IR-induced oxidative stress and altered antioxidant response have been extensively demonstrated in various in vitro and in vivo experimental models, the oxidative stress and modulation of the cellular antioxidant system as a bystander response to irradiation is much less-studied, and the available data are quite controversial. Several studies suggested that oxidative damage increased in bystander cells [2,40,62,63], and antioxidants were able to inhibit this bystander effect [64]. For instance, it was reported that nonirradiated bystander cells exposed to DNA fragments isolated from the medium [90] or to the medium itself [91] of low-dose- (0.1 Gy and 0.084 Gy, respectively) irradiated cells showed an increased intracellular generation of ROS. Khan et al. reported that partial lung irradiation with 10 Gy induced bystander effects (manifested in a higher micronuclei frequency) in the nonirradiated part of the lung, which could be inhibited with SOD [92]. Other in vitro works found that bystander cells cocultured with cells irradiated with a low (0.1 Gy) dose of high linear energy transfer (LET) radiation [5] or high doses (2 or 5 Gy) of low- [93] or high-LET radiation [5] showed higher levels of DNA damage, protein carbonylation and lipid peroxidation and reduced activity of the antioxidant enzymes.

In contrast to these previous findings, we could not demonstrate a higher level of oxidative damage in bystander animals 24 h after treatment with EVs from mice irradiated with either low or high doses of low-LET radiation. Similar to our results, El-Din et al. showed that lipid peroxidation in the spleen increased after whole-body irradiation with 2-Gy X-rays, but it did not change after localised (cranial) irradiation with 2 Gy, indicating the lack of bystander oxidative changes [83]. Interestingly, our data also showed that mice injected with EVs from either nonirradiated or irradiated mice had elevated levels of plasma oxidative damage as compared to control (0 Gy) mice. EVs contain various enzymes related to redox processes [66,67,68,69,70], oxidised DNA fragments [71,72] and even ROS [68,73]. We may hypothesise that, after intravenous injection with EVs, these molecules could be released from disintegrated EVs, thus causing an elevated level of ROMs in the circulation. More in vivo studies are needed to test this hypothesis.

On the other hand, we showed a significant decrease in the expression of multiple antioxidant enzyme genes (SOD2, CAT and GST) in the spleen of bystander mice treated with EVs originating from mice irradiated with 2 Gy. This result indicates a strong bystander response mediated by EVs influencing the antioxidant system of EV-recipient splenocytes. Similar to our findings, Przybyszewski et al. demonstrated that SOD2 and GPx activity strongly declined in irradiated human melanoma cells after 5-Gy γ-rays, as well as in cocultured bystander cells 24 h after the start of the experiment [93]. As far as we know, our study is the first that investigated the potential role of EVs in the transmission of radiation-induced bystander oxidative stress and the regulation of antioxidant enzyme expression in vivo.

Oxidative stress is a common promoter of apoptosis and cellular senescence [94,95,96,97]. Senescence arrests the proliferation of cells at risk for neoplastic transformation, while apoptosis is a programmed cell death in order to remove damaged or dysfunctional cells from the tissue [98]. P16 is a recognised senescence marker [53,98,99,100], which, in our study, showed strongly upregulated gene expression in the spleen 24 h after irradiation with 2 Gy, indicating a potential senescence induction in splenocytes after high-dose irradiation. This also correlates with our finding of reduced SOD2 expression in mice irradiated with 2 Gy, since SOD2 deficiency was shown to induce cellular senescence and increase p16 protein levels [101]. Some in vitro studies demonstrated that senescent cells released a greater number of EVs than control cells, and EVs from senescent cells (probably through the transmitted miRNAs) were able to activate senescence in nearby cells [102,103]. However, in vivo studies have not yet examined this potential bystander effect. In our in vivo study, we could not demonstrate the presence of senescence induction based on the p16 gene expression in the spleen 24 h after EV transfer from irradiated animals. Though, this result does not exclude the development of senescence at different time points after EV treatment.

An increased BBC3 gene expression detected both after low and high doses points to apoptosis induction, which is an expected finding after direct spleen irradiation and supports several previous studies [28,83,88,104,105]. Similar to our results, Li et al. found that, in murine blood cells, BBC3 expression was upregulated 24 h after exposure to 2-Gy γ-rays [42]. A very strong increase in the expression of the CDKN1A gene was observed in blood cells 8–48 h after 2–8-Gy irradiation in a murine study [42,59] and 6 h after 1.5-Gy irradiation in a human study [106], and our work also supports these findings. However, we detected no significant modification in the expression of BBC3 or CDKN1A genes in the spleens of bystander mice. In accordance with our results, Szatmári et al. found that, although the fraction of apoptotic cells in the spleen increased 24 h after 2-Gy irradiation, the EV transfer did not have an apoptosis-inducing effect in bystander spleen cells [28]. Similarly, Furlong et al. reported that proapoptotic and initiator caspase genes were upregulated one or 24 h after low-dose (0.05 Gy and 0.5 Gy) exposure in keratinocytes and in bystander cells as well, but effector caspases 3 and 7 genes were downregulated in bystander cells, not executing the apoptotic pathway to the final stages of cell death [107]. In contrast, in rats exposed to a 2-Gy dose of cranial irradiation, increases in late-apoptotic cells and necrotic cells were observed in the spleen 24 h after exposure [83]. The cranial irradiation of mice with 1-Gy X-rays also led to altered levels of cellular proliferation and apoptosis in bystander spleen tissues six hours and four days after exposure [19]. An in vitro study showed that increases in the number of apoptotic cells was observed in human keratinocytes 48 h after a medium transfer from 0.5-Gy- or 5-Gy-irradiated cells [14]. It is possible that initiation of the upregulation of genes connected to cell cycle regulation and the progression and termination of the apoptotic cascade was apparent in bystander mice at different time points than the examined 24 h or only in specific subpopulations of spleen cells.

For GADD45a, we observed a marginally significant decline 24 h after 2-Gy irradiation, similar to other studies that also found its decreased expression or no significant change in white blood cells after IR [42,106,108,109], and the EV transfer in our system could not initiate significant GADD45a gene expression changes in bystander splenocytes. Regarding ATM expression, it was reported to be downregulated in murine blood cells 24 h after exposure to 2-Gy γ-ray irradiation [42], but we found a significant increase after low-dose irradiation and no change after high-dose irradiation in spleen cells. Similar to our findings, exposure to 2-Gy γ-rays also did not alter the ATM expression in vitro measured 1–24 h after exposure [50].

**Figure 7:**
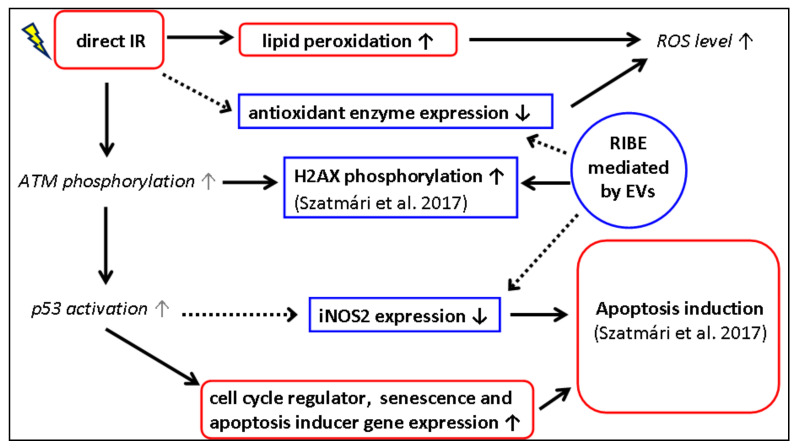
Schematic representation of the suggested pathways in mouse spleen cells detected 24 h after exposure to direct 2-Gy irradiation or to EVs originating from 2-Gy-irradiated mice. Texts in bold are the results presented in this study or in our previous study [28]. Solid-line arrows show an inducing effect, while dotted-line arrows show an inhibiting effect. Effects not studied in our work are shown in italics. Red boxes with rounded corners represent effects induced solely by direct irradiation, while blue boxes show effects induced by both direct irradiation and EV transmission.

We observed a decreased expression of iNOS2 both in 2-Gy-irradiated mice and in bystander mice receiving EVs from mice irradiated with 2 Gy. iNOS2 produces large amounts of NO after its induction, which can promote or inhibit programmed cell death in a variety of cell types, including T cells and splenic B-lymphocytes [110,111]. A possible mechanism for how IR influences the iNOS2 gene expression is via the DNA damage response pathway. Exposure to high-dose IR elicits higher ATM activation [64,112,113], which leads to higher γ-H2AX foci frequency [114,115] and increased p53 tumour-suppressor protein activation [116,117,118]. It was confirmed that p53 represses iNOS2 gene expression [119]. It is possible that EV-transmitted factors (most probably miRNAs) regulated specific genes post-transcriptionally, causing H2AX phosphorylation [31] and, also, the modified expression of antioxidant enzymes and iNOS2 in spleen cells (Figure 7).

It was suggested that the downregulation of antioxidants stabilise ROS production, which is important for apoptosis and senescence induction [88,101,120,121], while a reduced iNOS2 expression also contributes to higher apoptosis induction [110]. It is possible that EV-mediated factors (e.g., miRNAs and transcription factors) induce the downregulation of antioxidant enzyme genes and iNOS2 in order to prepare the tissue for programmed cell death in the case of excess harmful stimuli. MiRNAs can suppress gene expression through inhibition of the translation or degradation of targeted mRNAs [122,123], and we found several differently expressed miRNAs involved in pathways related to oxidative stress resistance, cell cycle regulation and apoptosis in the EVs of irradiated mice [28]. Further studies are needed to elucidate whether the observed bystander effect was the consequence of the transfer of specific miRNAs via the EVs.

## 5. Conclusions

In conclusion, our results show that EVs from mice irradiated with 2 Gy can alter the splenic cellular antioxidant system of naïve mice in a bystander manner by reducing the expression of antioxidant enzyme genes and genes connected to the cellular redox system (e.g., iNOS2). However, our data do not support other reports that RIBE induces elevated levels of oxidative stress. To our knowledge, ours is the first study to demonstrate that EVs originating from irradiated animals could induce these effects in vivo in bystander animals. Currently, it is still unclear whether bystander effects are beneficial, natural defence mechanisms triggered by stressors and mediated by compounds originating from stressed cells or, rather, if they induce deleterious modifications in recipient cells. It is possible that exposure to EVs from stressed cells leads to a higher level of stress in recipient bystander cells, but it also induces more resistance and robustness against subsequent stress, making the bystander effect an adaptive response [61,124,125,126]. Understanding the molecular mechanisms and signalling pathways of bystander effects and the exact role of EVs in this process is important for a better understanding and estimation of cancer risks and other health consequences associated with ionising radiation exposure.

## Data Availability

The data are presented within the paper. Additional raw data are available on request from the corresponding author.

## Supplementary Materials

The following are available online at https://www.mdpi.com/2076-3921/10/2/156/s1: Figure S1. Agarose gel electrophoresis (2% agarose) of PCR amplicons of the genes tested. Table S1. Gene descriptions and sequences of the forward (F) and reverse (R) oligonucleotid primers of the analysed genes.
