# Supplementary material for: Oxidative Stress and Gene Expression Modifications Mediated by Extracellular Vesicles: An In Vivo Study of the Radiation-Induced Bystander Effect
Source: Antioxidants (Basel). 2021 Jan 21;10(2):156. doi: 10.3390/antiox10020156 (PMC7911176; doi:10.3390/antiox10020156)
Supplement: Supplementary file 1 [file antioxidants-10-00156-s001.pdf]

Supplementary Material

Figure 1.
(A)

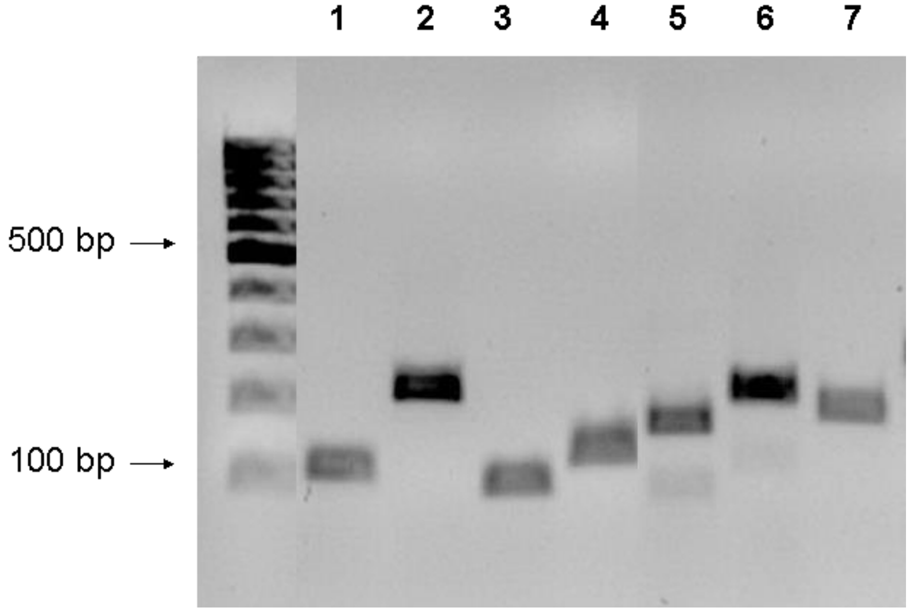

(B)

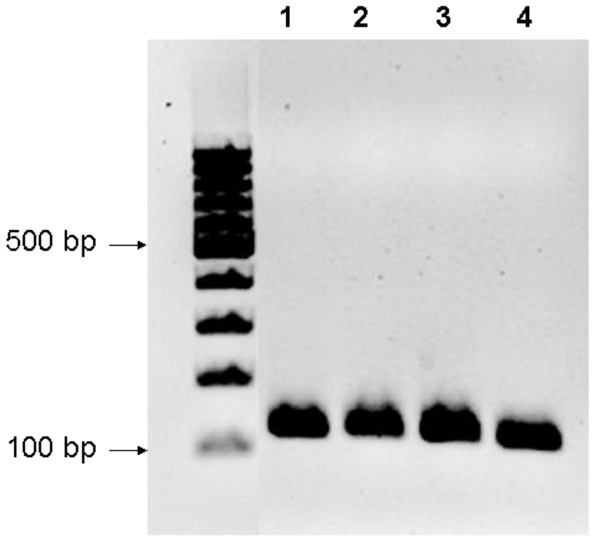

(C)

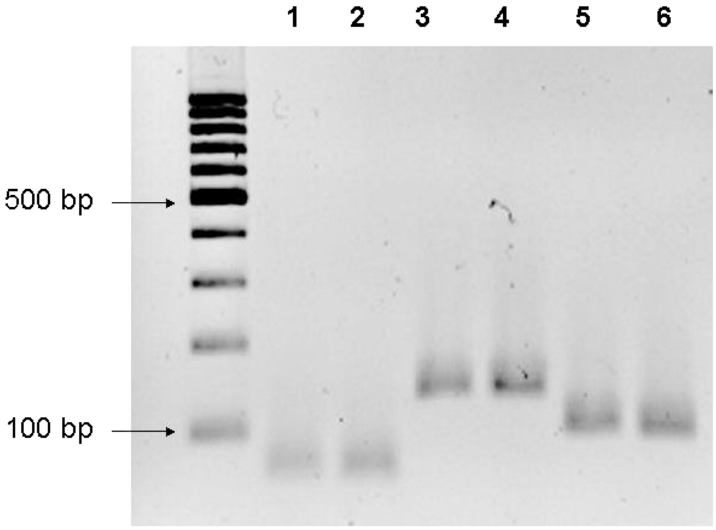

Figure 1. Agarose gel electrophoresis (2% agarose) of PCR amplicons of the genes tested. The expected amplicon size is shown in the parentheses. (A) Lane 1: COX-2 (125 bp), lane 2: SOD2 (245 bp), lane 3: GST (112 bp), lane 4: CAT (140 bp), lane 5: iNOS2 (173 bp), lane 6: p16 (215 bp) and lane 7: POLR2G (200 bp). (B) Lanes 1-4: BBC3 (122 bp). (C) Lanes 1-2: CDKN1A (66 bp), lanes 3-4: ATM (148 bp) and lanes 5-6: GADD45a (106 bp). We used a GeneRuler 100bp DNA ladder (Thermo Scientific Waltham, MA, USA).

Table 1. Gene descriptions and sequences of the forward (F) and reverse (R) oligonucleotid primers of the analysed genes.

Short gene name	Primer sequence 5'- 3'	Product size (bp)	Description
POLR2G	F TGTGGTCACTCAGGTCAACAAGGT<br>R TGTCTACACGCGTGCCTACAATCT	200	polymerase (RNA) II (DNA directed) polypeptide G; housekeeping gene
COX-2	F AGCCCATTGAACCTGGACTG<br>R ACCCAATCAGCGTTTCTCGT	125	prostaglandin-endoperoxide synthase 2; pro-inflammatory enzyme
SOD2	F GCTTACAGATTGCTGCCTGC<br>R TATAAACCAGCCCGGAGCCT	245	superoxide dismutase 2, mitochondrial; antioxidant enzyme
GST	F GTACACAGAAGGAGTGGCGG<br>R ACGGTTCCTGGTTTGTTCCT	112	glutathione S-transferase; antioxidant enzyme
CAT	F GTGCCCCCAACTATTACCCC<br>R GAATGTCCGCACCTGAGTGA	140	catalase; antioxidant enzyme
iNOS2	F CCTGCTTTGTGCGAAGTGTC<br>R CCCAAACACCAAGCTCATGC	173	nitric oxide synthase 2, inducible; pro- or antioxidant, apoptosis regulator
p16	F CTGCTCAACTACGGTGCAGA<br>R CCAGCGGAACGCAAATATCG	215	cyclin dependent kinase inhibitor 2A (Cdkn2a); cell-cycle regulator, senescence inducer
BBC3	F GACCTCAACGCGCAGTACGA<br>R GCTCCAGGATCCCTGGGTAA	122	BCL2 binding component 3; apoptosis inducer

CDKN1A	F GTCCCACTTTGCCAGCAGAATAA<br>R GGTCGGACATCACCAGGATTG	66	cyclin-dependent kinase inhibitor 1A (P21);<br>cell cycle regulator
ATM	F GCACACGGATTGCTCAAGGA<br>R GCCCATTCGGAATATGGATCAG	148	ataxia telangiectasia mutated;<br>DNA damage repair inducer
GADD45a	F CCTGCACTGTGTGCTGGTGA<br>R CCACTGATCCATGTAGCGACTTTC	106	growth arrest and DNA-damage-inducible 45 alpha;<br>DNA damage repair inducer, cell cycle regulator
